# Face and content validity of a holistic assessment questionnaire to assess cancer-related fatigue after breast cancer

**DOI:** 10.1080/21641846.2024.2389007

**Published:** 2024-08-18

**Authors:** Kim A.E. Wijlens, Annemieke Witteveen, Lian Beenhakker, Ester J.M. Siemerink, Reinoud Achterkamp, Sabine Siesling, Miriam M.R. Vollenbroek-Hutten, Christina Bode

**Affiliations:** a Department of Biomedical Signals and Systems, Technical Medical Centre, University of Twente, Enschede, The Netherlands; b Department of Internal Medicine, ZiekenhuisGroep Twente, Hengelo, The Netherlands; c Department of Oncology Rehabilitation and Pain, Roessingh Rehabilitation Center, Enschede, The Netherlands; d Department of Health Technology and Services Research, Technical Medical Centre, University of Twente, Enschede, The Netherlands; e Department of Research and Development, Netherlands Comprehensive Cancer Organisation (IKNL), Utrecht, The Netherlands; f Medisch Spectrum Twente, Enschede, The Netherlands; g Department of Psychology, Health and Technology, University of Twente, Enschede, The Netherlands

**Keywords:** Holistic monitoring, personalized care, mixed method, survivorship, cancer-related fatigue

## Abstract

**Background and objective:**

Cancer-related fatigue (CRF) affects the quality of life after breast cancer. In a previous study, we developed a 72-item questionnaire that assesses CRF from a holistic point of view; named the Holistic Assessment of CRF (HA-CRF) questionnaire. The current study assessed the face and content validity of the HA-CRF questionnaire.

**Methods:**

Using a mixed-method approach, ten breast cancer survivors (BCS) did a cognitive walkthrough of the HA-CRF via an app followed by a semi-structured interview about relevancy and essentiality (qualitative). In addition, ten health care professionals (HCPs) assessed the relevancy, clarity, and essentiality of each item via a questionnaire (quantitative).

**Results:**

BCS indicated minor textual improvement for four items and six items were not completely clear. The app was considered easy to use and the HC-CRF was on average completed in 18 minutes. The HA-CRF questionnaire provided openness about fatigue and gave the feeling of being heard. The items were helpful and induced self-awareness. HCPs indicated 71% of items being very clear or minor revisions proposed by the minority, with 64% of items being essential and 92% considered relevant.

**Conclusions:**

The HA-CRF showed good face and excellent content validity. Further research is needed to assess its ability to monitor in daily life.

## List of acronyms

BCS: Breast cancer survivors
BMS: Behavioural, Management and Social Sciences
CERQ: Cognitive Emotion Regulation Questionnaire
CMO: in Dutch ‘Commissie Mensgebonden Onderzoek’
CRF: Cancer-related fatigue
CVI: Content Validity Index
CVR: Content Validity Ratio
GSLTPAQ: Godin-Shephard Leisure-Time Physical Activity Questionnaire
HA-CRF: Holistic Assessment of CRF questionnaire
HCPs: Healthcare professionals
HDI: Helen Dowling Institute
HRQoL: Health Related Quality of Life
I-CVI: Content Validity Index - item score
IPAQ: International Physical Activity Questionnaire
KWF: Queen Wilhelmina Fund
*M*
: Mean
*n*
: number of BCS
NCCN: National Comprehensive Cancer Network
NWO: Dutch Research Council
PARTNR: Personalized cAnceR TreatmeNt and caRe
S-CVI: Content Validity Index – scale score
S-CVI/Ave: Content Validity Index – scale score average method
S-CVI/UA: Content Validity Index – scale score universal agreement method
SD: Standard deviation
STROBE: Strengthening the Reporting of Observational Studies in Epidemiology
TIIM: Twente Intervention and Interaction Machine
ZGT: in Dutch ‘Ziekenhuisgroep Twente’

## Introduction

Up to ten years after breast cancer diagnosis, one in five patients reports extreme fatigued [1]. Cancer-related fatigue (CRF) is defined by the National Comprehensive Cancer Network (NCCN) clinical practice guideline in oncology as ‘*a distressing, persistent, subjective sense of physical, emotional and / or cognitive tiredness or exhaustion related to cancer or cancer treatment that is not proportional to recent activity and interferes with usual functioning’* [2]. Patients’ health related quality of life (HRQoL) is affected by CRF as higher levels of CRF are associated with lower levels of functioning, general quality of life, and general mental health [3–5]. CRF experienced years after cancer treatment is unlikely to decrease without treatment, as perpetuating factors like insufficient coping with the experience CRF, dysfunctional CRF cognitions, fear of breast cancer recurrence, dysregulation of activities and sleep, and negative social interactions and low social support are responsible for the persistence of fatigue [6–15]. Several types of treatment are available but not each treatment showed clinically relevant improvement for each cancer survivor [16, 17]. As an example, breast cancer survivors (BCS) with unhelpful coping strategies might be more responsive to cognitive–behavioural therapy approaches compared to BCS with physical deconditioning [18]. There is no standard treatment that is most appropriate for the individual BCS [19]. These examples emphasize the need to tailor treatments [17]. Dekkers et al. [20] used self-reports to develop patient profiles with the aim to tailor care. To our knowledge, currently, no Dutch assessment tools for CRF exist that also incorporate perpetuating factors to tailor care. Therefore, we developed a Dutch Holistic Assessment of CRF questionnaire (HA-CRF) with breast cancer patients and survivors, and healthcare professionals (HCPs) working in the secondary and third line of care [21], resulting in a 72-item questionnaire consisting of four themes: experience of CRF, and the perpetuating factors day pattern, social health, and coping [22]. For day pattern and social health, onboarding items are included that adaptively lead to deepening items if relevant, based on the given answer. With regard to coping and experience of CRF, all items are considered relevant. For day pattern items two assessment modes might be possible: via an app or via a wearable [23]. In the Netherlands, 45% of healthcare users used an up for self-help and 25% a wearable to monitor health values according to a recent report by the Dutch National Institute for Public Health and the Environment about the state of digital care [24]. The use frequency declines with age and increases with educational level. Depending on the feasibility, day pattern items might be complemented or replaced by wearable data.

The developed HA-CRF requires validation as the holistic approach of the multidimensional CRF resulted in including items of 21 questionnaires instead of combining complete validated questionnaires [22, 25, 26]. Validated questionnaires provided items for multiple themes (e.g. day pattern and social health), therefore, it is not possible to combine generic and specific instruments to holistically assess CRF [22]. Besides, items were adapted to suit the target population [22]. Regarding questionnaire validation, content, face, construct, and convergent validation provide insight into the psychometric properties of a questionnaire [26–31]. Content validity refers to investigating whether the items address all the relevant aspects of the construct [26]. With face validity, it is researched if the questionnaire appears to measure as intended [27, 29]. Construct validity refers to the concept that is the objective of the study and the extent to which the questionnaire accurately assesses what it is supposed to [28]. Convergent validity indicates the correlation of the questionnaire with questionnaires that have similar concepts [27, 29]. Due to the holistic perspective, it is expected that it is particularly challenging to find relevant questionnaires with the same and different constructs or (sub)scales regarding construct and convergent validation [22]. Therefore, the goal of this study was to determine the face and content validity of the HA-CRF questionnaire [26, 27]. To determine content validity, the relevancy, clarity, and essentiality of the HA-CRF questionnaire were investigated using both qualitative and quantitative methods to validate the questionnaire [32–34].

## Method

### Study design

A combination of expert and target population judgment is advised to assess face and content validity [28]. The target population should evaluate whether the appropriate items are selected through cognitive interviewing and the expert population should quantitatively assess content validity [28]. Content validity evaluation by experts can be performed through quantified assessment allowing for statistical procedures or via the Delphi method [28]. Concerning experts, relevant stakeholders included HCPs working in secondary or tertiary line of care either in the field of breast cancer or cancer-related fatigue treatment. As it is challenging to arrange a group interview to conduct the Delphi method, quantify assessment was preferred. BCS experiencing fatigue were considered as the target population to assess whether the questionnaires measure CRF and whether it is feasible to complete via a smartphone app. In this mixed-method approach, a cognitive walkthrough of the HA-CRF questionnaire [22] (qualitative method) with ten BCS followed by a semi-structured interview and HCPs quantitatively assess the content validity. Cognitive walkthrough is a technique to assess whether items are understood, and clear [35]. During the semi-structured interviews, use, clarity, relevancy, and essentiality were assessed. The interview questions are reported in Table 5 (Supplementary Information A). The HA-CRF questionnaire is composed of several themes and elements which are illustrated in Table 1 and referred to in the results section. In the current study, all participants completed both screening and deepening items so that the validity of each item was assessed. For activity, onboarding items 18–20 adaptively leads to deepening items 24–31 based on the given answer to obtain a more thorough understanding. Item 23 is the onboarding item for sleep, with items 32–37 as deepening items. With regard to social, items 45 and 46 are the onboarding items and 47–51 are the deepening items. Item 39 has two versions that depend on the answer of item 38. When BCS are able to work, item 39 is related to their work being fulfilling. Item 39 is about their willingness to work when they indicated being unable to work in item 38. This HA-CRF questionnaire will in the future be used to personalize treatment advice. Using a smartphone app (Twente Intervention and Interaction Machine [TIIM]) for the HA-CRF questionnaire allows to use decision rules to provide personalized treatment advice in daily life.

**Table 1.** HA-CRF questionnaire items corresponding to the CRF construct’s themes and elements.

CRF themes	CRF elements	HA-CRF items
Experience of CRF		1–17
	Physical CRF	1–5
	Cognitive CRF	6–10
	Emotional CRF	11–15
Day pattern		18–37
	Activity	18–20 → 24–31
	Intentional behaviour	21–22
	Sleep	23 → 32–37
Social health		38–51
	Work	38–39
	Family	40–44
	Social	45, 46 → 47–51
Coping		52–71
	General	52
	Catastrophize	53–54
	Fear of cancer recurrence	55
	Optimism	56
	Insufficient coping with cancer	57–58
	Stressors	59–65
	Putting into perspective	66
	Dysfunctional cognitions	67–69
	Boundaries	70–71

→ indicates the onboarding items adaptively leading to deepening items.

Ethical approval of the study was given from all relevant bodies: Committee on Research involving human subjects (CMO, in Dutch ‘Commissie Mensgebonden Onderzoek’) Arnhem-Nijmegen, the clinical institute that provided the BCS (‘Ziekenhuisgroep Twente’ [ZGT] hospital), and the University of Twente (Faculty of Electrical Engineering, Mathematics and Computer Science). Informed consent was obtained from all participants (BCS and HCPs). The study was performed in accordance with the ethical standards as laid down in the 1964 Declaration of Helsinki and its later amendments.

For reporting, the ‘Strengthening the Reporting of Observational Studies in Epidemiology’ (STROBE) statement for cross-sectional studies was used where possible [36, 37]. Table 7 contains the full checklist (see Supplementary Information B).

### Participants

The patient information brochure and informed consent were sent to the BCS before a scheduled outpatient visit at the ZGT hospital. During their visit, BCS was asked if they were willing to participate directly thereafter. The cognitive walkthrough combined with the semi-structured interview was aimed to be within one hour and conducted by the first author (KW). Inclusion criteria were (1) being female, (2) diagnosed with breast cancer and treated with a curative intent irrespective of the year of diagnosis, (3) at least 18 years of age, (4) under follow-up care at ZGT, (5) able to read and write in the Dutch language, (6) willing and able to participate, and (7) able to provide informed consent. BCS were excluded if they (1) were also diagnosed with a different cancer than breast cancer, (2) had a relapse or metastases. Ten BCS participated between April and May 2022. Of the initially approached BCS, 91% participated. One BCS was excluded due to scheduling problems. Saturation was expected after five to ten BCS.

After the cognitive walkthroughs and interviews, HCPs were invited to quantitatively evaluate the content validity to complement the BCS perspective with the HCP perspective. HCPs of three clinical institutes were approached through members of the Personalized cAnceR TreatmeNt and caRe (PARTNR) project. The PARTNR project aims to develop an intelligent self-learning system for individuals with breast cancer to provide personalized treatment advice for cancer-related fatigue. The HCPs work in the field of breast cancer treatment (ZGT) or psychological treatment of CRF (Helen Dowling Institute, [HDI]), and rehabilitation (Roessingh Rehabilitation Centre). KW informed the HCPs about the interviews via email with the information letter. Sample size calculation for quantitative content validity is arbitrary; ten participants are recommended as optimum [34]. Therefore, ten HCPs were approached and participated between January and March 2023.

### Data collection

During the cognitive walkthrough, BCS were instructed to think aloud (indicate what they noticed or thought) while completing the four themes of the HA-CRF questionnaire (experience of CRF, day pattern, social health, and coping). The HA-CRF questionnaire was completed on a smartphone as it was implemented in the TIIM app. TIIM is a software application created by the BMS (Behavioural, Management and Social Sciences) Lab of the University of Twente. KW emphasized that the cognitive walkthrough was about testing the questionnaire instead of the BCS, therefore all suggestions were considered informative, and it was impossible to say something wrong. Notes were taken for adjustment or clarification of the questionnaire items. The duration for each part is an indication of the time that was needed to complete the questionnaire. Additionally, audio was recorded and collected data were summarised per item. Users should be able to complete the questionnaire via a smartphone app, therefore, BCS scored their experience with smartphone use using self-administered items, see Table 6 in Supplementary Information A. BCS indicated their experience from beginner to expert or as having no experience. In addition, to assess their experience with health monitoring through apps or wearables, BCS scored their experience with monitoring health via smartphones and wearables. The intended use of the HA-CRF questionnaire is to monitor CRF and provide personalized treatment advice, therefore, the BCS is asked about the variability of the themes and their desired frequency of use.

The content validity by HCPs was assessed quantitatively, administered through a questionnaire implemented in Qualtrics, assessing the relevancy (1), clarity (2), and essentiality (3) of each item with regard to the provided description, see Table 8 of Supplementary Information C [32–34]. For each theme and element of the HA-CRF questionnaire, a description was composed and discussed with co-authors AW and CB. For the element social the description was; low social support and negative social interactions perpetuate fatigue (when participants are not satisfied with their social life, deepening questions need to be answered).

### Data analysis

Clarity (1) of individual items was calculated using ten HCPs ratings on a 3-point Likert Scale from not clear to very clear. The clarity is summed per item and divided by the sample size resulting in a score between 1 and 3. Concerning essentiality (2), the content validity ratio (CVR) was calculated per item to assess which items were essential. The CVR is calculated by the number of respondents that scored the items essential (Ne) using the formula: (Ne-*n*/2)/(*n*/2) with *n* being our sample size of 10 [34]. CVR ranges between −1 and 1. Items that are not essential have a CVR < 0.6 (this value is based on the total number of experts, and the numerical values of the Lawshe Table 2) [38]. From relevancy questions (3) the content validity index (CVI) was calculated per item (I-CVI). To calculate I-CVI, the number of respondents that scored the item as quite or very relevant is summed and divided by the sample size (*n* = 10). CVI scores range between 0 and 1. Each item was judged according to Zamanzadeh et al. [34], who regarded items with a score of >0.79 as appropriate to include. Items scored between 0.70 and 0.79 need revision and below 0.70 should be eliminated. For the total questionnaire, a scale score (S) can be calculated (S-CVI), based on the average (S-CVI/Ave) or on the universal agreement method (S-CVI/UA) [33, 39]. The proportion of items for which all HCPs provided a score of 3 or 4 is considered the S-CVI/UA. The S-CVI/Ave is the average of all I-CVIs. Scores of 0.8 (S-CVI/UA) and 0.9 (S-CVI/Ave) or higher are considered excellent content validity [40]. Polit et al. [39] prefer the S-CVI/Ave, as the universal agreement method is extremely strict when there are many experts.

**Table 2.** Characteristics of breast cancer survivors (BCS), with *n* the number of BCS.

Characteristics	Breast cancer survivors (*n* = 10)					
Age [*M* (SD)]	52.4 (7.5)					
**Household composition**						
Living with a partner	8 (80%)					
Children at home (<14)	1 (10%)					
**Education**						
Low, middle^1^	8 (80%)					
High^2^	2 (20%)					
**Experience with**	Beginner	Intermediate	Practiced	Very practiced	Expert	No experience
Smartphone use	0 (0%)	1 (10%)	5 (50%)	2 (20%)	2 (20%)	0 (0%)
Monitoring health via smartphone (e.g. Google Fit / Apple health)	1 (10%)*	0 (0%)	2 (20%)	2 (20%)	1 (10%)	3 (30%)*
Monitoring health via wearables (e.g. Fitbit)	1 (10%)*	0 (0%)	2 (20%)	0 (0%)	1 (10%)	5 (50%)*
**Duration of questions**	CRF	Day pattern	Social health	Coping	Total questionnaire	Total interview duration
*M* [SD] in minutes	03:36 [00:48]	08:21 [01:49]	02:35 [00:33]	04:02 [00:33]	19:06 [03:04]	54:48 [11:05]

*n = *number of BCS, *M = *mean, SD* =* standard deviation, ^1^Primary and secondary education, ^2^Higher professional education or college/university. Experience is filled in on the following scale: beginner, intermediate, practiced, very practiced, expert and no experience. *One BCS indicated both beginner and no experience.

## Results

### Cognitive walkthrough and interviews

The characteristics of the BCS are shown in Table 2. BCS had a mean age of 52.4 years (standard deviation [SD] = 7.5 years). Most BCS were living with a partner, without children, and followed at least secondary education. Most BCS consider themselves practicing with a smartphone but have less experience with health monitoring. BCS is less experienced with wearables such as Fitbit. The cognitive walkthrough combined with semi-structured interview had a mean duration of 58:48 min (SD = 11 min).

The comments of the BCS during the cognitive walkthrough are described per the theme of the HA-CRF questionnaire.

Regarding the experience of CRF, item 7 [*How often did your fatigue make it difficult to organize your thoughts when doing things at work (include work at home)?*] concerns work situation, where two BCS commented: ‘*I don’t work*’ and ‘*I don’t work fulltime yet*’. Item 17 [*How many days were you tired during the past week*] concerned the number of fatigued days, on which the following comments were reported: ‘*not a single day, I was just tired for a moment, not an entire day*’ and ‘*yes, but never entire days*’.

For the day pattern, many BCS read the activity (activity levels in the past week) and behaviour response to fatigue items twice before answering. They indicated that the activity items were difficult to answer as they required a combination of activity duration, intensity, and frequency. One BCS suggested ‘*a diary does provide insight, insight in activity pattern*’ to replace the activity items. Item 21 [*Do you tend to do a lot on a good day and rest on a bad day?]* consist of two components, which caused confusion: ‘*Partly, I do too much on a good day but do not rest on a bad day. I answer sometimes as it is partially not applicable*’. Regarding sleep, one BCS chose the wrong answer for item 33 [*Do you stay awake all day without dozing]* filled in often as she often took naps instead of often staying awake without naps. Another BCS suggested the following adaptation to avoid confusion: ‘*Do you take naps during the day*’. Item 34 [*Are you asleep between 0200 and 0400?*] includes a time frame that made it difficult to interpret and suggestions for adaptation were provided: ‘*Are you asleep from 02.00 till 04.00 at night?*’.

For the theme social health, item 46 [*Do you think you are receiving sufficient support?*] has a dichotomic answer option instead of 4-point Likert, a BCS commented: ‘*That is actually somewhere in between. Thus ‘no’*’.

In the theme coping, one BCS read item 57 [*Do you think that things will be okay, no matter how your fatigue may change*?] three times and commented: ‘*difficult, fatigue changes but this item does not change, then I think I will be fine*’. Item 60 [*Do you realise how precious life is and make the most of it, since your cancer diagnosis and experiencing fatigue?*] consists of two components making it difficult to answer: ‘*Do you realize that life is valuable, and do you get the most out of it since you have cancer and are tired, I try several things but yeah … *’. With regard to peer support, one patient indicated she already has peer support which was not an answer option of item 63 [*Would you like to make contact with others in the same boat?*]. Lastly, item 66 [*Has your fatigue not been that bad compared to other things?*] required rephrasing: ‘*what other things?*’. The adaptation of items is described in Table 9 (Supplementary Information D).

During the semi-structed interviews, the use of the HA-CRF in the TIIM app was easy for 90% of the BCS and one BCS (10%) had a neutral opinion. The BCS appreciated that only one item was displayed on the screen and the four themes made it clear what to expect from the items. The duration to complete the HA-CRF was acceptable. One patient indicated ‘*it might be interesting to know whether their current activity level is as they desire*’. With regard to the clarity, items needed to be read thoroughly. In addition, the different aspects (how many times, more than 15 min, for three activity levels) of the activity items made them difficult to answer. Including household tasks as activity examples would make it more tangible. Concerning relevancy, BCS indicated that the HA-CRF questionnaire is composed of relevant items and also includes items they did not think of themselves, such as ‘*Do you know how to indicate your limits?*’ and ‘*Do you try to carry on your life as you’ve always done?*’. BCS indicated that some items might overlap with their breast cancer care instruments, such as with items of the Distress Thermometer. Others indicated similar items with regard to aspects of their revalidation treatment for CRF. Concluding, the TIIM app was rated nice, clear, easy to use, clarifying, neat, and not having too many aspects and the HA-CRF questionnaire was considered complete.

To determine the frequency of use, BCS was asked how often the different themes change and the desired frequency to fill in the questionnaire (e.g. weekly, monthly, yearly). BCS indicated that the experience of CRF can vary daily but prefers to fill it in weekly or less often. The variability of day patterns depends on their rhythm or energy and can be as variable as the experience of CRF. The variability of social health depends on work reintegration and probably stabilizes around two years after diagnosis for the majority of survivors due to the Dutch sick leave regulations. Coping with fatigue might be something that takes lots of time to change and as a patient indicated ‘*the answers will be comparable nine out of ten times*’. In addition, BCS mentioned that the frequency of filling in the items was related to the variability of the different themes and depended on the stage of their patient journey (e.g. within a year after breast cancer treatment, reintegrating at work). BCS preferred a higher frequency, for example weekly, shortly after they completed breast cancer treatment. However, years after breast cancer treatment the situation stabilizes unless you participate in CRF treatment. Therefore, the frequency to fill in the HA-CRF questionnaire should be based on the preference of the BCS keeping in mind the variability of her life.

BCS indicated that this HA-CRF questionnaire acknowledged fatigue and provided the feeling of being heard. The items were helpful for BCS themselves as they induce self-awareness, one BCS realized ‘*I need to listen to my body sooner*’. Others wanted to use the HA-CRF questionnaire to do something about their fatigue.

### Quantitative content validity

The characteristics of the ten HCPs from multiple disciplines working in the secondary and tertiary line of care institutes are shown in Table 3. The HCPs had a mean age of 46.5 years (SD = 10.8 years) with a mean work experience of 14.6 years (SD = 8.9 years).

**Table 3.** Characteristics of ten HCPs.

Profession (*n*)	Physician (2)	Specialized nurse (2)	Psychologist (3)	Paramedic^1^ (3)	Total (10)
Age [*M* (SD)] in years	48.5 (6.5)	58.5 (0.5)	33.0 (2.2)	50.7 (9.7)	46.5 (10.8)
Work experience [*M* (SD)] in years	13.8 (1.3)	14.0 (8.0)	7.3 (3.3)	22.7 (9.2)	14.6 (8.9)

*n = *number of HCPs, *M* = mean, and SD* = *standard deviation. ^1^Two social workers, one psychomotor therapist.

The clarity, CVR, and CVI scores are shown in Table 4 and described in the sections below.

**Table 4.** Quantitative content validity of the healthcare professionals (HCPs).

Clarity [1,3]	*#* items
3.0	15
2.9	17
2.8	19
2.7	4
2.6	10
2.5	7
CVR [−1,1]	*#* items
1.0	9
0.8	16
0.6	21
0.4	5
0.2	11
0	3
−0.2	6
−0.4	1
CVI [0,1]	*#* items
1.0	40
0.9	21
0.8	5
0.7	4
0.6	2

CVR = Content validity ratio. CVI = Content validity index. *#* = number. [] indicates the range.

#### Clarity

Average clarity scores for individual items ranged from 2.5 to 3.0 (see Table 4), with a median of 2.8. The deepening items of activity and the experience of CRF items were the least clear as respectively 75% (6/8) and 41% (7/17) of these items had a score of 2.5 or 2.6. Item 60 was adapted as an HCP indicating that the item contains two parts (*Do you realise how precious life is and make the most of it)*. To avoid double-barrelled items, item 60 was split into two items.

#### Essentiality (CVR)

64% of the items (46/72) were marked essential with a median of 0.6, see Table 4. Nonessential items can be eliminated, Table 9 (Supplementary Information D) includes a description of the eight items that were adapted and the eighteen items that remained unchanged. As an example, item 62 [*Do you have difficulties in believing that the fatigue happened to you?*] was eliminated as it was nonessential and required elimination as indicated by the CVI score. 46% of nonessential items (12/26) were deepening items of the day pattern. Experience of fatigue contains five nonessential items (about feeling exhausted, have energy, being forgetful, slowed down in thinking, and feeling helpless) distributed over the three CRF dimensions. For social health, only item 39 [*Is your work fulfilling?*] was considered nonessential. Regarding coping, seven items were considered nonessential concerning insufficient coping with cancer (items 57 and 58 [*Are you better able to accept the way things work out?]*), stressors (items 60, 62 and 63), and putting into perspective (item 66).

#### Relevancy (CVI)

The I-CVIs ranged from 0.6 to 1.0, Sixty-six items (92%) were considered relevant, for four items (6%) revisions were needed, and for two items (2%) elimination was advised, see Table 4. The items that needed revision or elimination concern sleep items (32 [*Are you satisfied with your sleep?]*, 34, 35 [*Do you spend less than 30 min awake at nights?*]), emotional fatigue [*Did you feel helpless?*], and coping with stressors (62, 63), see Table 9 (Supplementary Information D). Item 62 was eliminated as it was also considered nonessential. The S-CVI/UA was 0.54 and the S-CVI/Ave 0.93, indicating excellent content validity according to the average approach. For the universal agreement method, the content validity is below the acceptability criteria.

## Discussion

This study aimed to determine the face and content validity of the HA-CRF questionnaire with BCS and HCPs. To improve the HA-CRF questionnaire, small textual changes were made based on suggestions from BCS and HCPs. Both BCS and HCPs preferred to change double-barrelled items. Next to this, most day pattern items were considered nonessential by HCPs and difficult to answer by BCS. Furthermore, BCS struggled to answer the activity items of the day pattern because of the different aspects. An activity diary was the only suggested alternative. As a result of the mixed-method approach, HCPs quantified 92% of items relevant, 64% of items essential, and most items were clear for the majority of the construct’s aspects. BCS indicated that all items were relevant to assess fatigue.

### Strengths and limitations

Our study has several strengths. The intended use of the questionnaire is to monitor CRF after breast cancer in daily life by using a smartphone app, through which BCS additionally receives personalized treatment advice. The intended use emphasises the importance of validating the HA-CRF questionnaire with both experts (HCPs) as well as the target populations (BCS) as for several CRF treatments a patient requires a referral from an HCP. With the involved stakeholders, we assured that HCPs were included from a hospital as well as from tertiary line of care institutes that provide CRF treatments. The sample size of HCPs was sufficient to assess the content validity as the results show a resemblance between HCPs without outliers. The results of the HPCs correspond to the BCS. All BCS were recruited from one hospital since saturation was expected between five and ten BCS. To obtain a heterogeneous sample, BCS with high and low education but also diversity in age were included. Saturation was achieved as no substantial information was gathered in the last interview. The variance in education levels of the included BCS corresponds to those in the Dutch population [41]. Most Dutch women are diagnosed with breast cancer at the age of 50–74 years [42], similar to our population. Due to the heterogeneous sample, we expect the results to be representative of CRF in breast cancer survivors.

It is important to also acknowledge the limitations of the study. The content validity according to the universal agreement method, scored below the acceptability criterion. However, as indicated by Polit et al. [39] the universal agreement is overly stringent with many experts and might be biased if one expert does not understand the task. In the revision suggestions, one HCP indicated their own interest that did not align with the construct description. As the revision suggestion indicated misalignment with the task, the universal method might be too conservative to assess CVI. Therefore, the average approach was used to assess content validity and resulted in excellent content validity.

### Future research

The HA-CRF questionnaire is well developed as it shows good face and excellent content validity according to the average approach method. Regarding future research, it should be determined if the onboarding items can act as criterion items for the deepening items by criterion validation before the items are eliminated as most nonessential and unclear items concern day patterns [26]. Sleep and activity can potentially also be monitored continuously via an app or wearable. As these wearable data correlate satisfactorily with questionnaire data for lung cancer patients, another possibility might be to complement the onboarding items with wearable data [23]. The experience with smartphone and monitoring the health of the participants in this study was comparable to the report about the Dutch State of digital care [24]. However, since 50% lack experience it might be better to consider the use of the wearable as optional. Further research is needed to determine if the day pattern items can be complemented or replaced by wearable data. The willingness for a certain frequency to fill in items relates to the variability of the themes and the patient journey stage as also indicated by Gentile et al. [43]. As such, the frequency should be based on the preference of the individual patient, keeping in mind the variability of BCS life and the themes of the questionnaire. Further research is needed to determine if the information collected with the HA-CRF questionnaire can serve as input to provide personalized care and to monitor CRF in daily life or during treatment. To be able to assess the reliability of the HA-CRF questionnaire to be used for monitoring and personalized treatment advice, the variability of the construct should be determined. The next steps concerning the validation of the questionnaire are to assess the criterion validity, usability, and feasibility of the HA-CRF questionnaire for its intended use.

## Conclusion

The HA-CRF questionnaire after breast cancer shows good face and excellent content validity assessed by both HCPs and BCS. In the future, the use of wearable data might complement or replace day pattern items. Further research on validity and reliability is needed to assess the psychometric properties of the HA-CRF questionnaire before implementation in daily practice.

## Supplementary Material

Supplemental Material

Supplemental Material

Supplemental Material

Supplemental Material

## Data Availability

The generated datasets and the HA-CRF questionnaire are available from the corresponding author on reasonable request in Dutch. During the research phase, the HA-CRF questionnaire is not yet publicly available.
