# Supplementary material for: Face and content validity of a holistic assessment questionnaire to assess cancer-related fatigue after breast cancer
Source: Fatigue. 2024 Aug 18;12(4):293–307. doi: 10.1080/21641846.2024.2389007 (PMC11404859; doi:10.1080/21641846.2024.2389007)
Supplement: Supplemental Material [file RFTG_A_2389007_SM1357.docx]

# Supplementary Information D

Table 9 Calculation of the I-CVI for relevancy and clarity for each item.

| Theme | Item | Clarity | Interpretation | CVR | Interpretation | I-CVI | Interpretation |
| --- | --- | --- | --- | --- | --- | --- | --- |
| Experience of CRF | Q1 | 2.8 | Clear | 1.0 | Essential | 0.9 | Relevant |
|  | Q2 | 2.5 | Clear | 0.2 | Not essential | 1.0 | Relevant |
|  | Q3 | 2.6 | Clear | 0.0 | Not essential | 1.0 | Relevant |
|  | Q4 | 2.9 | Clear | 0.6 | Essential | 1.0 | Relevant |
|  | Q5 | 2.9 | Clear | 1.0 | Essential | 1.0 | Relevant |
|  | Q6 | 2.5 | Clear | 0.6 | Essential | 0.9 | Relevant |
|  | Q7 | 2.7 | Clear | 0.6 | Essential | 0.9 | Relevant |
|  | Q8 | 2.5 | Clear | 0.2 | Not essential | 0.8 | Relevant |
|  | Q9 | 2.6 | Clear | 0.4 | Not essential | 1.0 | Relevant |
|  | Q10 | 2.9 | Clear | 0.8 | Essential | 1.0 | Relevant |
|  | Q11 | 2.9 | Clear | 0.6 | Essential | 0.9 | Relevant |
|  | Q12 | 2.9 | Clear | 0.6 | Essential | 1.0 | Relevant |
|  | Q13 | 2.5 | Clear | 0.2 | Not essential | 0.7 | Revision |
|  | Q14 | 2.8 | Clear | 0.6 | Essential | 0.9 | Relevant |
|  | Q15 | 2.6 | Clear | 0.6 | Essential | 1.0 | Relevant |
|  | Q16 | 2.9 | Clear | 0.8 | Essential | 1.0 | Relevant |
|  | Q17 | 2.8 | Clear | 0.6 | Essential | 1.0 | Relevant |
| Day pattern | Q18 | 2.8 | Clear | 0.4 | Not essential | 1.0 | Relevant |
|  | Q19 | 2.9 | Clear | 0.6 | Essential | 1.0 | Relevant |
|  | Q20 | 2.7 | Clear | 0.6 | Essential | 1.0 | Relevant |
|  | Q21 | 2.7 | Clear | 0.6 | Essential | 0.9 | Relevant |
|  | Q22 | 2.9 | Clear | 0.6 | Essential | 1.0 | Relevant |
|  | Q23 | 2.9 | Clear | 0.8 | Essential | 0.9 | Relevant |
|  | Q24 | 2.6 | Clear | -0.2 | Not essential | 0.8 | Relevant |
|  | Q25 | 2.8 | Clear | -0.2 | Not essential | 0.9 | Relevant |
|  | Q26 | 2.6 | Clear | 0.0 | Not essential | 0.9 | Relevant |
|  | Q27 | 2.8 | Clear | 0.2 | Not essential | 1.0 | Relevant |
|  | Q28 | 2.6 | Clear | 0.0 | Not essential | 0.9 | Relevant |
|  | Q29 | 2.6 | Clear | -0.2 | Not essential | 0.8 | Relevant |
|  | Q30 | 2.5 | Clear | 0.2 | Not essential | 0.9 | Relevant |
|  | Q31 | 2.6 | Clear | 0.4 | Not essential | 0.9 | Relevant |
|  | Q32 | 2.8 | Clear | 0.2 | Not essential | 0.7 | Revision |
|  | Q33 | 3.0 | Clear | 0.6 | Essential | 1.0 | Relevant |
|  | Q34 | 2.8 | Clear | -0.2 | Not essential | 0.6 | Elimination |
|  | Q35 | 2.9 | Clear | -0.2 | Not essential | 0.7 | Revision |
|  | Q36 | 2.8 | Clear | 0.2 | Not essential | 1.0 | Relevant |
|  | Q37 | 2.9 | Clear | 0.6 | Essential | 1.0 | Relevant |
| Social health | Q38 | 2.9 | Clear | 1.0 | Essential | 1.0 | Relevant |
|  | Q39 | 2.9 | Clear | 0.4 | Not essential | 0.9 | Relevant |
|  | Q39 | 2.8 | Clear | 0.8 | Essential | 1.0 | Relevant |
|  | Q40 | 2.9 | Clear | 0.8 | Essential | 1.0 | Relevant |
|  | Q41 | 2.8 | Clear | 0.6 | Essential | 1.0 | Relevant |
|  | Q42 | 2.8 | Clear | 0.8 | Essential | 0.9 | Relevant |
|  | Q43 | 3.0 | Clear | 0.8 | Essential | 0.9 | Relevant |
|  | Q44 | 2.8 | Clear | 0.6 | Essential | 1.0 | Relevant |
|  | Q45 | 3.0 | Clear | 0.6 | Essential | 1.0 | Relevant |

Table 9 (continued) Calculation of the I-CVI for relevancy and clarity for each item.

| Theme | Item | Clarity | Interpretation | CVR | Interpretation | I-CVI | Interpretation |
| --- | --- | --- | --- | --- | --- | --- | --- |
| Social health | Q46 | 3.0 | Clear | 1.0 | Essential | 1.0 | Relevant |
|  | Q47 | 2.7 | Clear | 1.0 | Essential | 1.0 | Relevant |
|  | Q48 | 3.0 | Clear | 0.8 | Essential | 1.0 | Relevant |
|  | Q49 | 3.0 | Clear | 1.0 | Essential | 1.0 | Relevant |
|  | Q50 | 3.0 | Clear | 0.8 | Essential | 1.0 | Relevant |
|  | Q51 | 3.0 | Clear | 1.0 | Essential | 1.0 | Relevant |
| Coping | Q52 | 3.0 | Clear | 0.8 | Essential | 0.9 | Relevant |
|  | Q53 | 2.8 | Clear | 0.8 | Essential | 1.0 | Relevant |
|  | Q54 | 3.0 | Clear | 0.8 | Essential | 1.0 | Relevant |
|  | Q55 | 2.9 | Clear | 1.0 | Essential | 1.0 | Relevant |
|  | Q56 | 2.8 | Clear | 0.6 | Essential | 0.9 | Relevant |
|  | Q57 | 2.5 | Clear | -0.2 | Not essential | 0.9 | Relevant |
|  | Q58 | 2.5 | Clear | 0.2 | Not essential | 0.8 | Relevant |
|  | Q59 | 2.8 | Clear | 0.6 | Essential | 0.9 | Relevant |
|  | Q60 | 2.6 | Clear | 0.2 | Not essential | 0.8 | Relevant |
|  | Q61 | 2.9 | Clear | 0.8 | Essential | 0.9 | Relevant |
|  | Q62 | 2.6 | Clear | -0.4 | Not essential | 0.6 | Elimination |
|  | Q63 | 3.0 | Clear | 0.2 | Not essential | 0.7 | Revision |
|  | Q64 | 2.9 | Clear | 0.8 | Essential | 1.0 | Relevant |
|  | Q65 | 3.0 | Clear | 0.6 | Essential | 1.0 | Relevant |
|  | Q66 | 3.0 | Clear | 0.2 | Not essential | 1.0 | Relevant |
|  | Q67 | 2.8 | Clear | 0.8 | Essential | 1.0 | Relevant |
|  | Q68 | 3.0 | Clear | 0.8 | Essential | 1.0 | Relevant |
|  | Q69 | 2.9 | Clear | 0.4 | Not essential | 1.0 | Relevant |
|  | Q70 | 2.8 | Clear | 1.0 | Essential | 1.0 | Relevant |
|  | Q71 | 3.0 | Clear | 0.6 | Essential | 0.9 | Relevant |

NOTE: Number of items considered relevant, essential, and clear by all experts, *n* = 10. Values are shown with one decimal. Essential if CVR >0.62. For I-CVI, items with >0.79 are relevant, 0.70-0.79 need revision, and <0.7 should be eliminated. Items highlighted in yellow were changed as result of face and/or content validity.

Explanation

### Adapted items

Experience of fatigue items 3, 7, and 13 were rewritten with synonyms or the order of words was changed to be more understandable for BCS. These three items are [*How often do you have energy?*], [*How often did your fatigue make it difficult to organize your thoughts when doing things at work (include work at home)?*], and [*Did you feel helpless?*]. For experience of fatigue, one word has been added to item 17 [*How many days during the past week, did you experience fatigue*] as suggested by a patient. Regarding the items about activity (items 18-20, based on Godin-Shephard Leisure-Time Physical Activity {GSLTPAQ} questionnaire [1]), several adaptations were made to make the items clearer without changing the content including the tense, and activity examples. Item 21 [*Do you tend to do a lot on a good day and rest on a bad day?*] (activity behaviour) was ambiguous as it included both doing too little and too much. As more BCS tend to be overactive, item 21 is now about doing too much. Item 38 [*Are you able to work (include work at home)?*] was based on a validated questionnaire but the language level was too difficult, and the item was therefore simplified. Item 46 [*Do you think you are receiving sufficient support?*] (social health – social) lacked information about whom provides this support. Item 57 [*Do you think that things will be okay, no matter how your fatigue may change*?] was too vague with regard to change in fatigue. Item 60 [*Do you realise how precious life is and make the most of it?*] (coping with fatigue) was ambiguous as it concerned two different aspects which is divided into two items. Item 62 [*Do you have difficulties in believing that the fatigue happened to you?*] (coping with fatigue) was removed as it was not essential. Item 63 [*Would you like to make contact with others in the same boat?*] (coping with fatigue) is about peer support, and a BCS indicated that an answer option missed, which has been added. For item 66 [*Has your fatigue not been that bad compared to other things?*] (coping – putting into perspective) another item (item 34 instead of 25) of the Cognitive Emotion Regulation Questionnaire (CERQ) [2] questionnaire was selected as replacement as the items wording was unclear.

### Unchanged items

As experience of fatigue items 2, 8, and 9 were clear and relevant, but not essential these items were not removed nor changed. These three items are [*Have you felt exhausted?*], [*How often did your fatigue make you feel slowed down in your thinking?*], and [*How often did your fatigue make you more forgetful?*]. Activity items 24 to 31 (based on International Physical Activity Questionnaire {IPAQ} [3]) were not changed as this is a complete validated questionnaire about activity. Criterion validation is needed to determine if items 18 to 20 can serve as screening items and 24 to 31 as deepening items. This validation is necessary before elimination of these items is considered. This is also applicable to item 23 as screening for items 32 to 37 (based on RU-SATED [4]) to assess sleep. Items 58 [*Are you better able to accept the way things work out?*] (coping with cancer) and 69 [*Are you able to set your boundaries?*] (coping – dysfunctional cognitions) were not essential for HCPs but are essential for BCS and were therefore not removed.

# References

[1] **Godin, G**. (2011). The Godin-Shephard Leisure-Time Physical Activity Questionnaire. The Health & Fitness Journal of Canada, 4(1), 18–22. doi.org:10.14288/hfjc.v4i1.82

[2] **Garnefski N, Kraaij V, Spinhoven P**. CERQ Handleiding voor het gebruik van de Cognitive Emotion Regulation Questionnaire. Een vragenlijst voor het meten van cognitieve copingstrategieën [Internet]. Zoetemeer; 2002 [cited 2022 Nov 25]. Available from: https://service.datec.nl/cerq/download.aspx.

[3] **International consensus group.** Internationale lichamelijke activiteiten vragenlijst korte versie [Internet]. 1998 [cited 2022 Jan 31]. Available from: https://meetinstrumentenzorg.nl/wp-content/uploads/instrumenten/IPAQ-korte-versie-meetinstr.pdf.

[4] **Micoulaud-Franchi J-A, Coelho J, Ouazzani Touhami K**, **et al.** The Sleep Prism of Health. Explaining Health Across the Sciences. Champ: Springer Nature Switzerland; 2020. p. 289–315.
