# Supplementary material for: Face and content validity of a holistic assessment questionnaire to assess cancer-related fatigue after breast cancer
Source: Fatigue. 2024 Aug 18;12(4):293–307. doi: 10.1080/21641846.2024.2389007 (PMC11404859; doi:10.1080/21641846.2024.2389007)
Supplement: Supplemental Material [file RFTG_A_2389007_SM9506.docx]

# Supplementary Information A

Table 5 Questions of semi-structured interview with BCS.

| 1. | Was the explanation clear? |
| --- | --- |
| 2. | On a scale of 1 to 5: The toolkit was easy to use |
| 3. | What is your opinion on the appearance? |
| 4. | What do you think of the time it takes to complete the toolkit? |
| 5. | Have you completed parts of this toolkit before? |
| 6. | Would you like to make a change? |
| 7. | Would you like to be addressed formal or informal? |
| 8. | What would be a reason for you to use the toolkit? |
| 9.& 10. | Per dimension, how often does this change and what is the preferred frequency? |
| 11. | Did you miss any questions? |
| 12. | What is your opinion on the four parts? |
| 13. | Would you use this more often? Or certain parts of it? |
| 14. | Do you have (other) suggestions / comments? |

Table 6 Self-administered items to assess the experience with smartphone use and health monitoring through apps or wearables.

| Please check one of the boxes to indicate your experience with | | | | | | |
| --- | --- | --- | --- | --- | --- | --- |
|  | Beginner | Intermediate | Practiced | Very practiced | Expert | No experience |
| Smartphone use |  |  |  |  |  |  |
| Monitoring health via smartphone (e.g., Google Fit or Apple health) |  |  |  |  |  |  |
| Monitoring health via wearables (e.g., Fitbit) |  |  |  |  |  |  |
