# Supplementary material for: Face and content validity of a holistic assessment questionnaire to assess cancer-related fatigue after breast cancer
Source: Fatigue. 2024 Aug 18;12(4):293–307. doi: 10.1080/21641846.2024.2389007 (PMC11404859; doi:10.1080/21641846.2024.2389007)
Supplement: Supplemental Material [file RFTG_A_2389007_SM9502.docx]

# Supplementary Information C

Table 8 Questions of the content validity questionnaire of health care professionals [32].

| 1. | Is this item clear? * | 1 = not clear;  2 = item needs some revision;  3 = very clear. |
| --- | --- | --- |
| 2. | How essential is this item? | 1 = not necessary;  2 = useful but not essential;  3 = essential. |
| 3. | How relevant is this item? | 1 = not relevant;  2 = somewhat relevant;  3 = quite relevant;  4 = very relevant. |

** A text box was available to provide revision suggestions.*
